# Aspirin Exacerbated Respiratory Disease

**DOI:** 10.1155/2012/473863

**Published:** 2012-08-14

**Authors:** Luis M. Teran, Stephen T. Holgate, Hae-Sim Park, Anthony P. Sampson

**Affiliations:** ^1^Department of Immunogenetics and Allergy, Instituto Nacional de Enfermedades Respiratorias, Mexico City, Mexico; ^2^Clinical & Experimental Sciences, University of Southampton Faculty of Medicine, Southampton, UK; ^3^Department of Allergy and Clinical Immunology, Ajou University School of Medicine, San-5, Woncheondong, Youngtonggu, Suwon 442-721, Republic of Korea

As guest editors of this special issue of the *Journal of Allergy*, we are pleased to be able to present a range of articles, both reviews and original research papers, on the important topic of aspirin-exacerbated respiratory disease (AERD). AERD is a synonym for the “aspirin triad” of asthma, nasal polyposis and sensitivity to aspirin originally described by Widal ninety years ago. AERD patients typically experience severe bronchoconstriction and/or rhinoconjunctival reactions to aspirin, and also to other non-steroidal anti-inflammatory drugs (NSAID), even those which they have not encountered previously [1]. The reactions reflect non-allergic hypersensitivity as many AERD patients are not atopic and the reactions to NSAIDs are not usually IgE-mediated. While the aspirin triad has been described as emerging typically in early middle age, aspirin-intolerance, as determined in bronchoprovocation studies, may be apparent in 21% of adult asthmatics and 5% of asthmatic children, suggesting that it is under-recognised as a factor in asthma episodes [2]. Even in the absence of NSAID ingestion, AERD patients have relatively severe chronic asthma with a high proportion requiring long-term oral corticosteroid therapy, representing an unmet need of poorly-controlled asthma [1]. Nasal polyposis in AERD can be severe and require recurrent surgery.

Many of the papers in this special issue hinge upon the key insight in the field of AERD made by the late Professor Andrzej Szczeklik of Krakow. His “cyclooxygenase theory” recognised that the ability of NSAIDs to trigger adverse reactions in AERD patients correlates with their potency as cyclooxygenase inhibitors [3]. He extended the theory with the realisation that arachidonic acid, the substrate of the cyclooxygenase (COX) pathway, is also the substrate of the 5-lipoxygenase (5-LO) pathway that generates the cysteinyl-leukotriene family of mediators. The most persistent anomaly described in AERD is that even in the absence of NSAID ingestion, the levels of cysteinyl-leukotrienes are constitutively elevated in the bronchoalveolar lavage fluid, sputum, exhaled breath condensate, nasal secretions, saliva, blood and urine of AERD patients compared to aspirin-tolerant individuals [4]. That the further triggering of cysteinyl-leukotriene synthesis is important in acute AERD was established by studies with leukotriene synthesis inhibitors and leukotriene receptor antagonists [5]. The central mystery in AERD remains why only these patients, and not others with comparably severe asthma, show acute adverse responses to NSAIDs. This special issue of *J. Allergy *includes papers from many of the leading laboratories and opinion leaders working on this question around the world.

In the review article by Dr Maria Garcia-Cruz and her colleagues in Mexico, AERD and the cyclooxygenase theory are discussed with special emphasis on rhinosinusitis, its diagnosis and its treatment. Pathophysiological mechanisms are reviewed including recent studies of cytokine and chemokine anomalies in AERD, particularly in relation to *Staphylococcus aureus* enterotoxins which may drive abnormal allergic responses. The theme of rhinosinusitis is extended by the wide-ranging review paper from the group of Prof. Meyer in Hamburg, writing with Dr Schäfer from Erlangen, who stress the life-threatening nature of AERD and survey its molecular mechanisms, treatment and future directions for research.

Genetic studies into AERD, including candidate gene approaches and genome-wide association studies, may throw light on the underlying pathological mechanisms or provide genetic biomarkers for improved diagnosis and treatment. The area is expertly reviewed by Dr Palikhe and colleagues based in Korea, while its importance is underlined by the original research presented by Dr Falfan-Valencia and colleagues in Mexico, describing a novel association between AERD and a polymorphism in the gene encoding the inflammatory cytokine interleukin-1*β*.

Over-production of cysteinyl-leukotrienes by LTC_4_ synthase and over-expression of the cysteinyl-leukotriene CysLT_1_ receptor have been postulated as central components of AERD pathology in the upper and lower airways [5]. The paper by Dr Steinke and his colleagues in Virginia, USA, provides a powerful argument that the Th2 cytokine interleukin-4 not only upregulates LTC_4_ synthase and CysLT_1_ receptors, but also down-regulates key components of the COX pathways, including COX-2, PGE_2_ synthase and the EP2 receptor, and that this accounts for the key features of AERD. They further outline a molecular mechanism by which IL-4 expression could itself be modulated by aspirin. Dr Jackson and colleagues from Southampton, UK, report that the allied Th2 cytokine IL-13 appears not to upregulate LTC_4_ synthase in human lung macrophages, perhaps supporting this unique role for IL-4 in AERD.

An enduring problem in the field of AERD is early and accurate diagnosis of the condition. Patients may not recognise an association between their use of NSAIDs and asthma exacerbations, while the gold standard for many years has been provocation testing using various oral or inhaled aspirin protocols that are not practicable in many clinical settings. The review in this issue by Dr Schäfer and Prof. Maune surveys the wide range of *in vitro* models that have been proposed as a basis for laboratory testing for NSAID sensitivity, including tests based on platelet aggregation, lymphocyte transformation, and others based on levels of eicosanoids and other mediators in serum or exhaled breath condensate. The original paper from Dr Abuaf and colleagues in Paris describes studies on a basophil activation test which shows promise in the diagnosis of AERD, at least in the sub-group of patients with greatest hypersensitivity.

 Finally, this special issue of *J. Allergy* includes a paper by Dr Mastalerz and colleagues from the group in Krakow, Poland, led by Prof Szczeklik. Based on liquid chromatography and mass spectrometry of exhaled breath condensates, this paper completes a broad survey of lipid anomalies in AERD, including prostanoids and novel products of the 5- and 15-lipoxygenase pathways. This is one of the last papers to bear the name of Andrzej Szczeklik as an author before his untimely death in February 2012, and this special issue of the *Journal of Allergy* is dedicated to his memory.

**A Remembrance of Professor Andrzej Szceklik (1938–2012)**

This special issue of the Journal of Allergy is privileged to include a paper (Mastalerz et al.) coauthored by Professor Andrzej Szczeklik of Krakow University shortly before his death, aged 73, on February 3rd this year. This paper is therefore one of Professor Szczeklik's last publications in a career in which he achieved high national and international honours and great scientific eminence for his founding insights and enduring contributions to the field of AERD. Andrzej Szczeklik was born in 1938 and graduated with a Diploma in Medicine from Krakow in 1961. His postgraduate training included work in Sweden at the Karolinska Institute and at Uppsala University, and in the USA at the University of North Carolina, Chapel Hill. After several years in Wroclaw, Poland, he returned to Krakow in 1972 and rose to become the Chairman of the Department of Medicine at the Jagiellonian University in 1989. From 1990 to 1993, he was president of the Copernican Academy and in 1993–1996 the Vice-Rector of Jagiellonian University Medical College. Szczeklik was well known by his scientific reputation to all the contributors to this special issue for his lifelong research into aspirin-intolerance. He led the international field following his insight in 1975 that aspirin intolerance arises from the pharmacological activity of aspirin and other NSAIDs in inhibiting prostaglandin synthesis by cyclooxygenase. The “cyclooxygenase theory” is the basis of much of the research focused on lipid mediators that followed in the field of AERD. Szczeklik's achievements were recognised by a plethora of awards and honours, including the Gold Medal of the Jagiellonian University, the AAAAI Robert A Cook Lectureship, memberships of the Polish Academy of Sciences, the Royal College of Physicians in London, and the Pontifical Academy of Sciences in the Vatican, and a number of visiting professorships and honorary doctorates at universities in the UK, Europe, and Japan. During the 1980s, he was active in the Solidarity Movement and remained a major contributor to the broader cultural life of his native Poland. Those who knew Andrzej as a friend will remember him not only as a gentleman and a scholar, but as an accomplished pianist and the author of three thoughtful and well-received books, “*Catharsis: The Art of Medicine”, “Kore”, and “Immortality: Promethean Dream of Medicine”* published posthumously, reflecting on a long life in medicine and the values that link the humanities and sciences. He will be sadly missed by his many scientific and clinical friends across the world.

*Luis M. Teran* *Luis M. Teran* *Stephen T. Holgate* *Stephen T. Holgate* *Hae-Sim Park* *Hae-Sim Park* *Anthony P. Sampson* *Anthony P. Sampson*
